# Correlation of Cognitive Status and Atrophy Score in Alzheimer’s Disease Among the Bangladeshi Population

**DOI:** 10.7759/cureus.65833

**Published:** 2024-07-31

**Authors:** Maliha Hakim, Mahmudul Islam, Mohammad Akter Hossain, Mohammad Nur Uddin, Murshed Baqui, Mashfiqul Hasan, Mohammad Nazrul Islam, Mim Tanzila Mamun, Alif Al Mamun, Redoy Ranjan, Md. Abdullah Yusuf, Ghulam Kawnayn

**Affiliations:** 1 Neurology, National Institute of Neurosciences and Hospital, Dhaka, BGD; 2 Neurology, Dhaka Medical College, Dhaka, BGD; 3 Endocrinology, Diabetes and Metabolism, National Institute of Neurosciences and Hospital, Dhaka, BGD; 4 Neuroradiology, National Institute of Neurosciences and Hospital, Dhaka, BGD; 5 Public Health, Central Queensland Hospital and Health Service, Queensland, AUS; 6 Cardiac Surgery, Bangabandhu Sheikh Mujib Medical University, Dhaka, BGD; 7 Biological Science, Royal Holloway University of London, London, GBR; 8 Microbiology, National Institute of Neurosciences and Hospital, Dhaka, BGD; 9 Neurology, Combined Military Hospital, Dhaka, BGD

**Keywords:** koedam’s score, medial temporal lobe atrophy (mta) scale, alzheimer’s disease mild cognitive impairment magnetic resonance imaging : neuropathology alzheimer’s disease mild cognitive impairment magnetic resonance imaging : neuropathology, early onset alzheimer's disease, alzheimer's disease

## Abstract

Background: Alzheimer’s disease (AD) patients suffer from cognitive dysfunction. This study assessed the structural magnetic resonance imaging (MRI) scoring among Alzheimer’s patients (age ≥18 years) to correlate with dementia severity according to mini-mental state exam (MMSE) scores.

Methods: This cross-sectional study evaluated Bangladeshi adult AD patients from January 2018 to December 2022 who attended with subjective memory complaints and fulfilled the diagnostic and statistical manual of mental disorders criteria (DSM 5) for diagnosing dementia. The medial temporal lobe atrophy (MTA) and Koedam’s score of the atrophy were measured utilising the 1.5 and 3 Tesla Magnetom symphony MRI systems.

Results: Of the 62 patients enrolled, the majority (39 cases; 62.9%) were aged over 60 years. Males were more predominant than females, with a male-to-female ratio of 2.6:1, and the moderate MMSE group consisted of 35.6% males and 64.7% females (P = 0.01). Further, MTA score severity is paradoxically associated with the MMSE score (P = 0.005). Additionally, we found a statistically significant negative correlation between the severity of the MMSE and only MTA scores (r = −0.350; 95% CI −0.551 to −0.110; P = 0.005).

Conclusion: Structural magnetic resonance imaging among Alzheimer’s patients is significantly correlated with the severity of dementia as per mini-mental state exam scores.

## Introduction

Dementia is a term used to describe symptoms of cognitive deterioration, which is multifactorial and common in the elderly population [1,2]. The two most common types of dementia among its multiple subtypes are Alzheimer's disease (AD) and vascular cognitive impairment (VCI). Dementias are subtyped according to their underlying pathologies, primarily defined by the accumulation of abnormal protein aggregates in neurons and glia, as well as in the extracellular compartment in certain regions of the brain [2-4]. Proteinopathies often exist years before clinical abnormalities develop. It is debatable whether these proteins are merely indicators that reflect the toxicity of the surrounding molecules, toxic agents themselves, or a combination of both [3-5].

Routine functional imaging may not be helpful for all dementia cases; however, imaging can exclude the treatable causes of dementia. A CT scan or MRI may immediately disclose an unsuspected frontal brain tumour, metastases, stroke, subdural hematoma, or hydrocephalus as the cause of dementia [4-6]. Moreover, imaging accurately demonstrates brain pathology in dementia disorders, including neoplastic infiltration in lymphomas and high-signal intensity on T2-weighted MR images in the pulvinar nucleus and medial thalamus in Creutzfeldt-Jakob disease [4,5]. In developing countries like Bangladesh, the MRI is the only way to correlate Alzheimer's disease with clinical findings. The MRI scores for diagnosing AD are critical, and medial temporal atrophy (MTA) is assessed using a five-point scale in coronal views on T1-weighted MRI sequences [6-8]. Evidence-based data have confirmed that visual evaluation of the anteromedial temporal lobe for atrophy on MRI has a sensitivity of 83.0% to 85.0% and a specificity of 96.0% to 98.0% to differentiate patients with AD from those without Alzheimer's disease [7-9].

The incidence of dementia patients is on the rise, coinciding with Bangladesh's increasing life expectancy, which now surpasses 70 years [10-12]. This has led to an increase in the number of dementia patients because this disease primarily affects the elderly age group. According to the latest WHO data published in 2020, the deaths due to Alzheimer's disease and dementia in Bangladesh have reached 14,993, or 2.09% of total deaths [13-15]. The age-adjusted death rate is 13.89 per 100,000 population, and Bangladesh ranks 142 in the world [10,14].

This study assessed the structural magnetic resonance imaging (MRI) scores among Alzheimer's patients to observe the correlation between dementia severity and the mini-mental state exam (MMSE) scores.

## Materials and methods

Study design and population

This analytical cross-sectional study observed adult AD patients at the National Institute of Neurosciences and Hospital (NINS&H), Bangladesh, from January 2019 to December 2022. This study included all patients aged ≥18 years, both male and female, who were attended to with subjective memory complaints and fulfilled the criteria for diagnosing dementia as outlined in the Diagnostic and Statistical Manual of Mental Disorders (DSM-5; fifth edition). The patients were recruited purposively, and consecutive patients were included in this study reporting to the dementia clinic. To exclude bias, other neurodegenerative dementias and different reversible causes of dementia, like brain tumours, infections, head injuries, substance abuse, depression, and malnourished patients, were excluded from this study. The National Institute of Neurosciences and Hospital in Dhaka, Bangladesh, is the only referral centre for neurosciences in the country, where all dementia patients are referred. Thus, this study sample represents the disease status of the Bangladeshi population. All procedures of the present study were carried out according to the principles of the Declaration of Helsinki and the ethical guidelines of institutional research ethics. The NINS&H ethics committee granted formal ethical approval to conduct the study (IRB no. NINS/2023/312). All participants consented willingly to participate in the study during the data collection periods. All data were collected anonymously and encrypted.

Study procedure

The demographic profile details were recorded and evaluated per standard procedure in the dementia clinic, and the DSM-5 criteria were applied to diagnose dementia patients. Further, a MMSE was performed to score the severity of cognitive domains [16]. The MMSE tests have multiple cognitive domains like orientation, repetition, verbal recall, attention and calculation, language, and visual construction. Scores range from 0 to 30 points, with lower scores indicating more significant impairment. The maximum score for the MMSE is 30. MMSE scores of approximately 21 to 25 are consistent with mild dementia, 11 to 20 with moderate dementia, and 0 to 10 with severe dementia. This can vary according to the educational level of the patients. A score of 25 or higher was classified as usual. If the score was below 24, the result was usually considered abnormal, indicating possible cognitive impairment. When the MMSE score was below 25, the patients were regarded as a case of dementia.

To exclude the reversible causes of AD, we utilised different routine blood tests like blood sugar, serum creatinine, complete blood count, peripheral blood film, vitamin D, and thyroid function tests (thyroid stimulating hormone, free triiodothyronine 3 and 4 levels). The liver function test (LFT), like serum albumin, serum glutamic pyruvic transaminase and bilirubin, the Venereal Disease Research Laboratory, the Treponema Pallidum Hemagglutination Assay, serum vitamin B12, serum calcium, parathyroid hormone, and chest X-ray, were also advised to exclude different reversible causes of dementia. A neuroimaging test, like a computed tomography (CT) and MRI scan, was performed to exclude the non-reversible causes of dementia, like stroke. However, neurodegenerative causes of dementia were confirmed by MRI scans according to the dementia protocol. All these imaging tests were conducted in the Department of Neuroradiology and Imaging at the same institute using the same standard protocol.

MRI protocol for Alzheimer's disease

We used the Siemens 1.5 and 3 Tesla MRI systems, which have appropriately phased array coils and follow the MRI protocol [8]. Further, MR imaging sequences observe the structural changes in T1-weighted axial, sagittal, coronal, and thin coronal oblique image sequences. The atrophy rating was done using the MTA scale and Koedam's score [6,17]. Two experts (a neurologist and intervention radiologist) confirmed the diagnosis of AD using MRI imaging findings, who were unaware of the subjects' age and clinical diagnosis. Once both raters agreed, a definitive final score was given.

Medial temporal lobe atrophy

The medial temporal lobe atrophy (MTA) score was assessed on coronal TSE images, which have a consistent slice position, using the protocol published by Scheltens et al. [6]. This scale was developed using the five-point semi-quantitative scale using T1 MRI or CT scans. In the MTA scale, a score of "0" indicated no atrophy, a score of "1" indicated only choroid fissure widening, a score of "2" stated temporal horn of the lateral ventricle widening, a score of "3" indicated moderate loss of hippocampus volume and decrease in height, and a score of "4" indicated severe loss of the hippocampus volume. The hemispheres (right and left) were evaluated separately, and the MTA score was calculated as the average of these two scores. Again, the raw data were dichotomised, and scores of 0, 1, and 2 were considered harmful for significant atrophy, demonstrating only no or mild atrophy, while scores of 3 and 4 were considered positive for moderate-to-severe atrophy.

Koedam's Score or Posterior Atrophy (PA) Rating Scale

According to Koedam et al., a "0" score indicates no atrophy, a "1" score suggests mild sulci widening without evident volume loss of the gyri, a "2" score indicates substantial sulci widening and volume loss of the gyri, and a "3" score indicates severe end-stage atrophy [10]. If different scores were obtained, the higher one was used.

Statistical analysis

Statistical analysis was performed using SPSS, version 28.0 (IBM statistics for Windows, NY). Continuous variables were presented as mean ± standard deviation (SD), while categorical variables were presented as frequency counts and percentages. Further, the chi-square test was utilised to compare categorical variables, while the Student t-test was employed to compare continuous variables. All possible efforts were made to obtain missing data, and variations between the case and control groups were analysed. The correlation between atrophy scores and dementia severity was assessed using the Pearson correlation coefficient, as assessed by MMSE scores. A two-sided P value of <0.05 was considered to have statistical significance.

## Results

A total of 62 AD patients were evaluated, and males were more predominant than females, with a male-to-female ratio of 2.6:1 (Table 1). Most patients (62.9%) were in the age group of more than 60 years, of which 43.6% were in the moderate and only 10.3% were in the severe MMSE group. Furthermore, the illiterate group of the population has significantly higher moderate-to-severe MMSE scores compared to educated patients (P = 0.03).

**Table 1:** Baseline characteristics of the study participants. MMSE: Mini-Mental State Examination; P-value reached from Chi-square test.

Variables		MMSE group				Total	P-value
		Normal (>25)	Mild (21 to 25)	Moderate (11 to 20)	Severe (0 to 10)		
Age group	≤60 Years	3 (13.0%)	9 (39.1%)	10 (43.5%)	1 (4.3%)	23 (100.0%)	0.202
	>60 Years	11 (28.2%)	7 (17.9%)	17 (43.6%)	4 (10.3%)	39 (100.0%)	
Gender	Male	14 (31.1%)	13 (28.9%)	16 (35.6%)	2 (4.4%)	45 (100.0%)	0.012
	Female	0 (0.0%)	3 (17.6%)	11 (64.7%)	3 (17.6%)	17 (100.0%)	
Residence	Rural	5 (31.3%)	1 (6.3%)	7 (43.8%)	3 (18.8%)	16 (100.0%)	0.216
	Urban	5 (17.9%)	10 (35.7%)	11 (39.3%)	2 (7.1%)	28 (100.0%)	
	Town	4 (22.2%)	5 (27.8%)	9 (50.0%)	0 (0.0%)	18 (100.0%)	
Education	Illiterate	1 (7.7%)	1 (7.7%)	8 (61.5%)	3 (23.1%)	13 (100.0%)	0.032
	Primary	3 (15.0%)	7 (35.0%)	10 (50.0%)	0 (0.0%)	20 (100.0%)	
	Secondary	4 (22.2%)	6 (33.3%)	6 (33.3%)	2 (11.1%)	18 (100.0%)	
	Graduate	6 (54.5%)	2 (18.2%)	3 (27.3%)	0 (0.0%)	11 (100.0%)	
Socio-economic status	Low	2 (8.3%)	9 (37.5%)	10 (41.7%)	3 (12.5%)	24 (100.0%)	0.140
	Middle	9 (27.3%)	6 (18.2%)	16 (48.5%)	2 (6.1%)	33 (100.0%)	
	High	3 (60.0%)	1 (20.0%)	1 (20.0%)	0 (0.0%)	5 (100.0%)	

The severity of lobar atrophy was assessed using the MTA score and was found to be 1.2±1.05, 2.1±1.17 and 2.8±0.45 in the normal, mild, moderate and severe MMSE score groups (p=0.005). However, the difference in the Koedem score among the MMSE group was not statistically significant (p=0.133) (Table 2).

**Table 2:** Comparison of severity of temporal lobar atrophy scores with severity of mini-mental state examination (MMSE). Data expressed as mean ± SD; MTA: medial temporal lobe atrophy; one-way ANOVA test was performed to see the level of significance. P <0.005 is considered as significant.

MMSE score	Sample	MTA score	95% confidence interval	P-value
Mild (21 to 25)	16	1.2 ± 1.05	0.63–1.75	0.005
Moderate (11 to 20)	27	2.1 ± 1.17	1.61–2.54	
Severe (0 to 10)	5	2.8 ± 0.45	2.24–3.36	
MMSE score		Koedem score		
Mild (21 to 25)	16	0.4 ± 0.88	−0.10 to 0.85	0.133
Moderate (11 to 20)	27	0.6 ± 1.12	0.19–1.07	
Severe (0 to 10)	5	0.0 ± 0.00	0.00–0.00	

Considering the relationship between the MMSE score and MTA score, it was a negatively weak correlation (r = −0.350; 95% CI −0.551 to −0.110), which was statistically significant (p = 0.005) (Table 3). Additionally, the MMSE and Koedem score correlation was found to be an insignificant negative correlation (r = −0.233; 95% CI −0.456 to 0.018; P = 0.069). The MTA and Koedem scores also demonstrated an insignificant negative correlation (r = −0.014; 95% CI −0.262 to 0.237; P = 0.917).

**Table 3:** Correlation coefficient between the severities of lobar atrophy scores with severity of Mini-Mental State Examination (MMSE). MMSE: Mini-Mental State Examination; GCS: Global Cortical Atrophy scale, MTA: medial temporal lobe atrophy.

Different scale	Pearson correlation (r)	P-value	95% confidence intervals	
			Lower	Upper
MMSE score and MTA score	−0.350	0.005	−0.551	−0.110
MMSE score and Koedem score	−0.233	0.069	−0.456	0.018
MTA score and Koedem score	−0.014	0.917	−0.262	0.237

**Figure 1:**
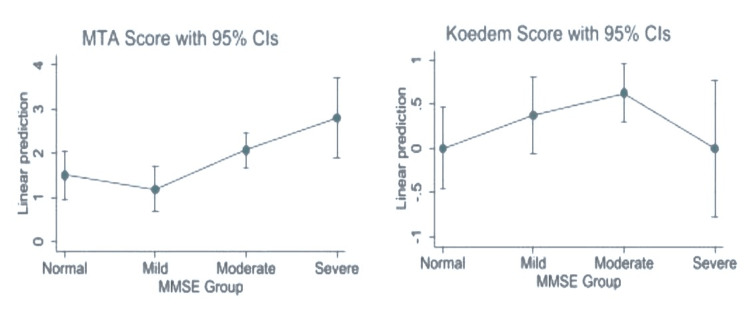
The Margins plot showing the adjusted prediction of different scale with MMSE score of the study population.

Furthermore, the Margins plot observed the linear predictive capabilities between the MMSE score and MTA score, whereas the Koedem score was found to have a sporadic correlation (Figure 1).

## Discussion

This study found that males predominantly suffered from AD, with a male-to-female ratio of 2.6:1, and the majority of the AD patients were over 60 years old. Further, we observed a significant negative correlation between the severity of the MMSE score and the MTA score (r = −0.350; P = 0.005).

Late-life dementia is a multifactorial pathology often attributed to Alzheimer's disease [18,19]. MRI imaging is used to quantitatively assess the extent, impact, and possible cause of regional brain pathology [2,6-9]. Existing research comparing MRI scores in clinically diagnosed Alzheimer's disease and vascular dementia patients was limited and inconclusive [3,7]. However, a recent study with larger sample sizes, comparing MRI differences between vascular dementia and Alzheimer's disease, suggests an explicit link between MTA, especially hippocampal atrophy, and clinical AD rather than vascular dementia [4]. Furthermore, hippocampal atrophy is the key feature in clinical Alzheimer's disease imaging and strongly correlates with Alzheimer's disease pathology [5-8]. Like our study results, several authors have also found a higher rate of MTA in well-characterised vascular dementia patients and its association with cognitive functioning, similar to other published papers [2,10,20].

Despite pathological differences, we found a significant correlation between the severity of MTA and MMSE scores, representing a potential association between Alzheimer's disease and vascular dementia populations supported by existing literature [21-23]. The MTA values were found to have a strong and equal association with cognition in AD and vascular dementia populations, explaining ~13% of the MMSE score variance. Conversely, vascular measures, especially white matter hyperintensities (WMH), were only associated with cognition in vascular dementia, and the MIRAGE study group explained a slight ~4% variance of the scores [21]. Nonetheless, WMH has been negatively correlated with cognitive functioning in individuals with normal cognition, mild cognitive impairment, vascular dementia, and AD, which suggests that the impact of this pathology extends beyond clinical diagnosis [2,24,25]. These findings indicate that neuronal cell loss is specific to cognition, independent of dementia aetiology, in concordance with published papers [26-28]. Although the patient's age is significantly associated with brain atrophy in both groups, the age of onset is more strongly related to vascular dementia than in Alzheimer's disease patients [24,25]. This study finding suggests that Alzheimer's disease pathology seems to overshadow age-related differences, while normal ageing and vascular disease interplay in vascular dementia [10,15,29].

The research has a few limitations that need to be acknowledged. The small sample size and cross-sectional design limit the study's ability to assess individual atrophy rates and predict the transition from a non-demented state to a demented state. However, considering the rarity and lack of evidence from the Bangladeshi perspective on AD, the current study findings added valuable insights to the literature. Calculating MMSE, MTA, and Koedem scores could also introduce bias; however, the severity of the scores was confirmed by two experts (neurologists and radiologists), which helps mitigate the risk of bias. Furthermore, using semi-quantitative measures may reduce sensitivity to group differences; however, having a single rater for MRI-based atrophy and cerebrovascular health scales presents a unique opportunity to observe the correlation between MMSE and atrophy scores [30].

## Conclusions

Bangladeshi males predominantly had Alzheimer's disease compared to women at the age of 60. Additionally, severities of medial temporal lobe atrophy had a significant negative correlation with the severity of the Mini-Mental State Examination score. This study suggests that Alzheimer's disease pathology primarily contributes to age-related differences in cognitive decline, whereas normal ageing and vascular disease play a more interactive role in the development of vascular dementia.
